# Fulminant Chromobacterium violaceum Infection With Suspected Septic Arthritis and a Concurrent Dengue Virus Infection: A Case Report

**DOI:** 10.7759/cureus.108021

**Published:** 2026-04-30

**Authors:** Hasan A Al-Humairi, Alaa A Elhadi, Aisha O Al Ali, Mariam A Tmeeheer, Yasir A Al-Humairi

**Affiliations:** 1 College of Medicine, University of Sharjah, Sharjah, ARE; 2 Orthopaedics and Trauma, Rashid Hospital, Dubai, ARE

**Keywords:** case report, chromobacterium violaceum, dengue virus infection, fulminant sepsis, opportunistic infection, septic ankle arthritis

## Abstract

*Chromobacterium violaceum* is a rare but highly virulent Gram-negative organism found in tropical and subtropical environments, associated with rapidly progressive sepsis and high mortality. Musculoskeletal involvement, particularly septic arthritis, is exceedingly uncommon. We report a case of a previously healthy 23-year-old male who presented with progressive left ankle pain, swelling, and fever. Initial evaluation revealed thrombocytopenia, markedly elevated inflammatory markers, and positive serology for Dengue virus infection, suggestive of a possible concurrent infection. Blood cultures grew *C. violaceum*, confirming bacteremia. Despite initiation of intravenous antibiotics and supportive care, the patient experienced rapid clinical deterioration with septic shock, severe metabolic acidosis, hyperkalemia, and multi-organ failure. He required intubation and vasopressor support but suffered multiple cardiac arrests and died within three days of hospitalization.

This case highlights the aggressive clinical course of *C. violaceum* infection, even in immunocompetent individuals, and underscores the diagnostic and therapeutic challenges associated with this rare pathogen. Concurrent dengue infection may have contributed to immune dysregulation and worsened disease severity. Early recognition, prompt diagnosis, and appropriate antimicrobial therapy are critical in managing *C. violaceum* infections. Clinicians should maintain a high index of suspicion in patients presenting with rapidly progressive sepsis, especially in endemic regions or in the presence of potential environmental exposure.

## Introduction

*Chromobacterium violaceum* is considered a rare cause of morbidity and mortality, with around 200 cases reported in the literature. It is a Gram-negative, facultatively anaerobic bacillus that is commonly found in soil and stagnant water in tropical and subtropical regions [1]. *C. violaceum* was first isolated in animals in 1905, and in 1927, it was the first case reported in a human [2]. Humans infected with this rare bacterium suffer great morbidity and mortality due to the rapid progression to septicemia. Reported cases in the literature mostly involve skin and soft tissue infections following exposure to contaminated water or soil, commonly through breaks in the skin [2], which may subsequently progress to bacteremia or disseminated infection.

Musculoskeletal involvement due to *C. violaceum* is particularly rare, which poses a challenge when it comes to the diagnosis, especially with the rapid deterioration associated with the disease. Only a limited number of cases of septic arthritis caused by this organism have been described in the literature [2].

In this report, we describe a case of a young male presenting with clinically diagnosed septic arthritis caused by *C. violaceum* with a possible concurrent dengue virus infection, which deteriorates into a fatal outcome. We aim to show how the disease progresses rapidly and the importance of early recognition and prompt treatment.

## Case presentation

This is a case of a 23-year-old, previously healthy male who presented with bilateral foot swelling of five days’ duration. One week prior to the presentation, he developed pain in the left ankle, accompanied by mild swelling and redness. The symptoms developed gradually and progressively worsened over the following days, eventually limiting his ability to bear weight and walk. Before the onset of the left ankle symptoms, the patient reported pain and swelling of the right foot, which resolved spontaneously without medical intervention. He denied any preceding trauma or injury to the affected joints and reported no swelling in other joints at that time.

About 15 days before the presentation, the patient developed continuous fever accompanied by chills. He did not record his temperature at home. He reported taking paracetamol for symptom relief but was unsure whether he had taken any antibiotics during that period. The fever was associated with a mild dry cough, nausea, and occasional vomiting. The patient denied any other symptoms.

Approximately 12 days prior to presentation, he received an intramuscular injection of vitamin B complex in the gluteal region, which was administered at home with the assistance of his roommates.

The patient had no known chronic medical conditions and reported no drug allergies.

He lives in shared accommodation with approximately 10 roommates. He reported smoking four to six cigarettes daily, but stopped smoking 15 days prior to presentation. He denied alcohol consumption.

The patient had traveled from Pakistan 11 months before the presentation.

Upon examination, the patient was conscious, alert, and communicating but appeared clinically fatigued. He was febrile and tachycardic, though other vital signs were within normal limits. Examination of the chest revealed clear lung fields with equal air entry bilaterally, and cardiovascular examination demonstrated regular S1 and S2 heart sounds with a tachycardic rate. The abdomen was soft, lax, and non-tender.

**Figure 1:**
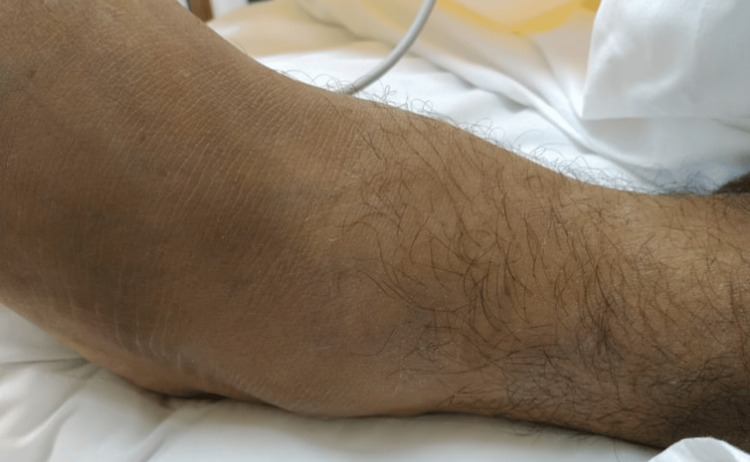
Left ankle swelling

Focused examination of the lower limb revealed a moderate swelling of the ankle with pitting edema (Figure 1). The overlying skin on the medial surface was erythematous and warm to the touch, raising suspicion for septic arthritis. There was no visible ulceration, discharge, or cutaneous injury noted at the time of admission. The patient was unable to bear weight with a painful range of motion in the left ankle.

During the acute deterioration on day 3 of admission, the patient was reassessed. He remained conscious and oriented, but was noted to be tachycardic, tachypneic, and had cold extremities. Peripheral oxygen saturation (SpO_2_) was unable to be recorded. Following intubation and sedation, the patient’s Glasgow Coma Scale (GCS) deteriorated to 3/15.

Blood work upon admission revealed a microcytic anemia, thrombocytopenia, and a normal leukocyte count with a left shift (neutrophils 81.0%). There was no relevant history or previous documentation of iron deficiency anemia or thalassemia that explains the previous findings. The inflammatory markers were markedly elevated (Tables 1, 2). Renal function and electrolytes were within normal limits. Serology was positive for dengue virus IgM and IgG antibodies, with non-structural protein 1 antigen (NS1 Ag) negative, while tests for HIV, malaria, SARS-CoV-2, and methicillin-resistant *Staphylococcus aureus* (MRSA) were negative.

**Table 1:** CBC -- Day 1 CBC: complete blood count; L: low (below reference range); H: high (above reference range).

Investigation	Result	Reference
White blood cell count (×10^3^/µL)	8.9	3.6-11.0
Red blood cell count (×10^6^/µL)	5.27	4.5-5.5
Hemoglobin (g/dL)	9.8 (L)	13.0-17.0
Hematocrit (%)	29.4 (L)	40.0-50.0
Mean corpuscular volume (fL)	55.8 (L)	77.0-95.0
Mean corpuscular hemoglobin (pg)	18.5 (L)	27.0-32.0
Mean corpuscular hemoglobin concentration (g/dL)	33.2	31.5-34.5
Red cell distribution width (%)	16.7 (H)	11.5-14.0
Platelet count (×10^3^/µL)	101 (L)	150-410
Neutrophils (×10^3^/µL)	7.21 (H)	2.00-7.00
Lymphocytes (×10^3^/µL)	0.53 (L)	1.00-3.00
Monocytes (×10^3^/µL)	1.07 (H)	0.20-1.00
Eosinophils (×10^3^/µL)	0.09	0.00-0.50
Basophils (×10^3^/µL)	0.00	0.00-0.10

**Table 2:** Inflammatory markers -- Day 1 CRP: C-reactive protein; H: high (above reference range).

Investigation	Result	Reference
CRP (mg/L)	191.6 (H)	<5.0
Procalcitonin (ng/mL)	2.15 (H)	<0.05

**Figure 2:**
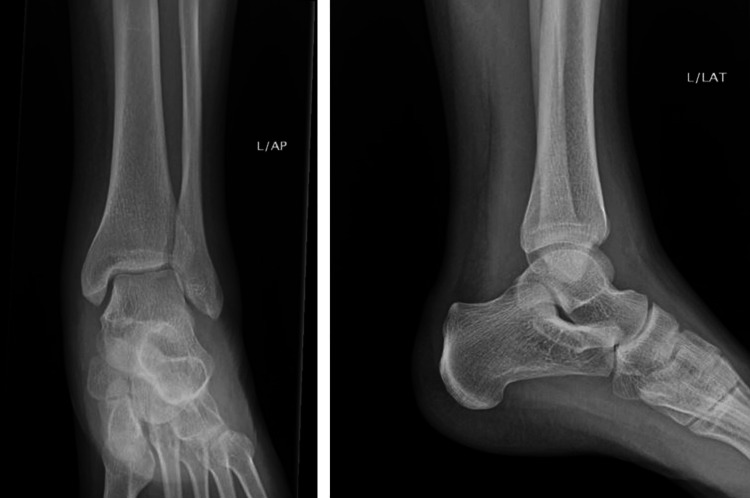
Left ankle X-rays anteroposterior (left) and lateral (right) Note that X-ray has a limited part in the diagnosis of septic arthritis, and further investigations are required. The diagnosis in this case remained clinical due to the rapid deterioration.

Given the severity of the left ankle swelling and erythema, an X-ray of the ankle was performed, which showed no evidence of fracture, dislocation, or osseous lesion (Figure 2). A concurrent chest X-ray was clear, with a normal cardiothoracic ratio.

Blood cultures drawn on admission grew Gram-negative rods after approximately 12 hours of incubation. The organism was subsequently identified as *C. violaceum* on the VITEK 2 system. Antimicrobial susceptibility testing was performed, and the results were interpreted according to the 2026 Clinical and Laboratory Standards Institute (CLSI) M100 36th edition guidelines for *Pseudomonas aeruginosa* [3], and it demonstrated susceptibility to cefepime, ceftazidime, ceftriaxone, gentamicin, and trimethoprim-sulfamethoxazole.

The results showed positive titers for both dengue-specific IgM and IgG antibodies. The presence of both antibodies, combined with a negative NS1 antigen result, is highly suggestive of a secondary dengue infection. The patient was diagnosed with septic arthritis of the left ankle secondary to *C. violaceum* bacteremia, with a concurrent dengue virus infection. 

The patient was initially admitted to the ward under the trauma surgery team for the management of septic arthritis and was co-managed with the Infectious Disease service. Empiric intravenous antibiotics were initiated, which included amoxicillin-clavulanate and vancomycin, and later switched to ceftriaxone. On day 2, the patient was given cefepime, gentamicin, and meropenem after susceptibility results returned. 

On day 3 of admission, the patient acutely deteriorated with respiratory distress and hemodynamic instability. The Rapid Response Team was activated, and the patient was urgently intubated by the anesthesia team and connected to mechanical ventilation (SIMV mode, FiO_2_ 100%). Following intubation, the patient suffered a cardiac arrest; return of spontaneous circulation (ROSC) was achieved after 7 minutes of cardiopulmonary resuscitation (CPR). The patient was transferred to the intensive care unit (ICU) on high-dose vasopressor support (noradrenaline) and sedation (fentanyl).

Despite maximal resuscitative efforts and ICU support, the patient's condition continued to decline. He developed multi-organ failure, including acute respiratory distress syndrome (ARDS), acute kidney injury, severe lactic acidosis, hyperkalemia, and disseminated intravascular coagulation (DIC), as evidenced by severely elevated prothrombin time (PT), activated partial thromboplastin time (APTT), and D-dimer (Tables 3-6). He suffered two further cardiac arrests during the early morning of day 3. Following the third arrest, ROSC could not be achieved, and the patient was declared dead at 05:35 on day 3 due to cardiac arrest following septicemia.

**Table 3:** ABG results during deterioration -- Day 3 *Laboratory artifact. ABG: arterial blood gas; pH: potential of hydrogen; pCO_2_: partial pressure of carbon dioxide; pO_2_: partial pressure of oxygen; HCO_3_: bicarbonate; SO_2_: oxygen saturation.

Investigation	At 01:30	At 04:59	Reference
pH	7.377	6.893	7.35-7.45
pCO_2_ (mmHg)	52.2	85.1	35-45
pO_2_ (mmHg)	102	61.0	83-108
HCO_3_ (mmol/L)	30.6	16.4	21-28
Lactate (mmol/L)	16	29	<2.0
Potassium (K+) (mmol/L)	8.5	8.7	3.3-4.8
Sodium (Na+) (mmol/L)	155	147	134-143
Ionized calcium (mmol/L)	0.84	2.58	1.15-1.29
Glucose (mg/dL)	-*	731	65-14
SO_2_ (%)	97.2	64.7	95-99

**Table 4:** Biochemistry -- Day 3 L: low (below reference range); H: high (above reference range).

Investigation	Result	Reference
Creatinine (mg/dL)	2.50 (H)	0.70-1.20
Magnesium (mg/dL)	4.6 (H)	1.6-2.6
Urea (mg/dL)	65 (H)	12-40
Sodium (mmol/L)	132 (L)	136-145
Potassium (mmol/L)	>10 (H)	3.3-4.8
Bicarbonate (HCO_3_) (mmol/L)	4.8 (L)	20-28

**Table 5:** Coagulation panel -- Day 3 PT: prothrombin time; INR: international normalized ratio; APTT: activated partial thromboplastin time; L: low (below reference range); H: high (above reference range).

Investigation	Result	Reference
PT (seconds)	17.1 (H)	11-14
INR	1.45 (H)	0.8-1.1
APTT (seconds)	44.9 (H)	28-44
D-Dimer (µg/mL FEU)	5.26 (H)	<0.5

**Table 6:** Urine routine -- Day 3 WBC/HPF: white blood cells per high-power field; RBC/HPF: red blood cells per high-power field.

Investigation	Result	Reference
Urine color	Yellow	Yellow
Urine clarity	Clear	Clear
Urine pH	5.5	5.0-7.5
Urine protein	1+	Negative
Urine glucose	Negative	Negative
Ketone	Negative	Negative
Nitrate	Negative	Negative
Blood by strip	1+	Negative
WBC/HPF	5-10	0-5
RBC/HPF	0-2	0-2
Epithelial cells/HPF	Not seen	Not seen
Mucus threads	Few	Not seen

## Discussion

*Chromobacterium violaceum* is an environmental Gram-negative bacillus found in soil and freshwater, predominantly in tropical and subtropical climates [4]. It is a rare but highly virulent bacterium and has a high mortality rate due to septic shock and multiorgan failure, especially in patients who are immunocompromised [5]. It produces the pigment violacein, which gives its colonies their characteristic violet color and is the basis for its name. The most common route of infection is typically a breakthrough of the skin when in contact with contaminated water or ingestion [4]. An oral route of infection may be considered in patients presenting with diarrhea as the sole symptom [6], while inhalational exposure has been implicated in cases of pneumonia [7], and direct inoculation is more consistent with localized soft tissue infection or cellulitis. *C. violaceum* can disseminate quickly from the initial site of infection, spreading via the bloodstream to internal organs and leading to multiple abscesses and sepsis [4,8]; this dissemination commonly involves the spleen, liver, and lungs, and more rarely the brain. Sepsis due to *C. violaceum* has been reported more frequently in individuals with underlying conditions such as chronic granulomatous disease, neutrophil dysfunction, and severe polymorphonuclear G6PD deficiency [9,10].

Bacterial sensitivity has been reported with agents such as co-trimoxazole and fluoroquinolones, including ciprofloxacin. In vitro data suggest that *C. violaceum* is also commonly sensitive to chloramphenicol, tetracyclines, and carbapenems. In contrast, it tends to demonstrate resistance to penicillin and narrow-spectrum cephalosporins, while responses to aminoglycosides and third-generation cephalosporins appear to be variable [11-13]. In this case, the source of infection was thought to be musculoskeletal, most likely septic arthritis, based on the presence of superficial erythema, left ankle swelling, and the patient’s inability to weight bear or mobilize. Despite being a previously healthy young man with no clear evidence of immunosuppression, he developed *C. violaceum* sepsis that progressed rapidly. This occurred despite early recognition and appropriate treatment, ultimately leading to significant multi-organ dysfunction and cardiac arrest, from which he did not recover.

Given the organism’s recognized tendency to cause abscess formation and disseminated infection, further invasive imaging and investigations, including CT of the chest, abdomen, and pelvis, with consideration of MRI of the brain, were planned to evaluate for metastatic foci and abscess formation; however, this was not possible due to his rapid clinical deterioration and inability to sustain life despite maximal supportive treatment.

Notably, the presence of both dengue virus IgM and IgG could suggest a recent infection, which may have further complicated the clinical course and contributed to the severity of the bacterial sepsis. Viral infections such as dengue are known to cause transient immune dysregulation, endothelial dysfunction, and increased vascular permeability, all of which may predispose to more severe secondary bacterial infections and a heightened inflammatory response [14]. However, there is a lack of firm evidence of whether this patient had a late primary infection or a secondary dengue infection contributing to the rapid and fatal course of the disease.

This case highlights the diagnostic challenge posed by *C. violaceum*, particularly in non-endemic settings where suspicion may be low. Early features are often non-specific, and standard empirical therapy for septic arthritis may be inadequate due to its resistance profile. Prompt microbiological identification and early escalation of care are therefore essential, especially in patients with rapidly progressive sepsis, even without typical risk factors.

Finally, this case emphasizes the need for a high index of suspicion for disseminated infection in patients with *C. violaceum* bacteremia. Where clinically feasible, early imaging to identify and control metastatic foci is essential, as source control remains a key determinant of outcome. Unfortunately, in this instance, the patient’s rapid deterioration precluded further diagnostic evaluation and definitive source control, reflecting the aggressive nature of this pathogen and its associated high mortality.

Limitations

This case has certain limitations. Due to the patient's extremely rapid clinical deterioration and subsequent hemodynamic instability, a diagnostic arthrocentesis of the left ankle was unable to be performed prior to the onset of septic shock. Consequently, the diagnosis of septic arthritis was based on clinical examination findings, including localized erythema, warmth, and significant swelling, rather than synovial fluid analysis. Additionally, while blood cultures confirmed *C. violaceum* bacteremia, the rapid progression to death precluded further advanced imaging, such as CT and MRI, to identify potential metastatic abscesses in other organs. Furthermore, testing for occult immunodeficiency was not done due to the rapid course of the disease, where neutrophil dysfunction, chronic granulomatous disease, or other conditions could have contributed to the outcome of this disease. 

Furthermore, the positive dengue serology does not confirm an active infection. Positive IgG and IgM and a negative NS1 Ag could reflect a late primary infection or a secondary dengue infection. There was no other supportive evidence available at the time.

## Conclusions

This case shows the fulminant course of *C. violaceum* infection with a clinically presumed musculoskeletal source. The presence of a possible concurrent dengue virus infection may have contributed to immune dysfunction, facilitating rapid progression to septic shock and multi-organ failure.

Early identification and timely initiation of appropriate antimicrobial therapy are essential, given the rapid dissemination of this organism. Clinicians should consider *C. violaceum* in the differential diagnosis of severe sepsis with unclear source, particularly in tropical and subtropical settings. Aggressive supportive management may improve outcomes, although prognosis remains poor in advanced disease.

This report reinforces the importance of clinical vigilance, early diagnosis, and awareness of rare but highly virulent pathogens in patients with rapidly deteriorating clinical courses.
